# Effects of corn grain processing and protein source on calf performance, rumen fermentation, and blood metabolites

**DOI:** 10.1038/s41598-023-37365-w

**Published:** 2023-06-22

**Authors:** M. Jafarpour, M. Alikhani, A. Riasi, H. Omidi-Mirzaei, M. Khorvash, M. H. Ghaffari

**Affiliations:** 1 Department of Animal Science, College of Agriculture, Isfahan University of Technology, Isfahan, 84156-83111 Iran; 2 Animal Science Research Department, Isfahan Agricultural and Natural Resources Research and Education Center, AREEO, Isfahan, Iran; 3 Institute of Animal Science, University of Bonn, 53111 Bonn, Germany

**Keywords:** Developmental biology, Physiology

## Abstract

The objective of this study was to investigate the effects of the interaction between corn grain processing and protein source on feed intake, growth performance, rumen fermentation, and blood metabolites of dairy calves. Seventy-two 3-day-old Holstein calves with an initial weight of 39.1 ± 3.24 kg were randomly assigned (n = 12 calves (6 male and 6 female) per treatment) to a 2 × 3 factorial arrangement of treatments with the factors of physical form of the corn grain [coarsely ground (CG) and steam-flaked (SF)] and protein type [canola meal (CAN), canola meal + soybean meal (CASY), and soybean meal (SOY)] were assigned. The study showed a significant correlation between corn grain processing method and protein source on calf performance, including starter feed intake, total dry matter intake (DMI), body weight, average daily gain (ADG), and feed efficiency (FE). The CG-CAN and SF-SOY treatments resulted in the highest feed intake and DMI in the post-weaning and total period, respectively. Interestingly, corn processing did not affect feed intake, ADG, and FE, but the highest ADG was observed at SF-SOY and CG-CAN. In addition, the interaction between corn processing method and protein source improved FE in calves fed CG-CAN and SF-SOY during the preweaning period and throughout the period. Although skeletal growth parameters were unchanged, calves fed SOY and CASY had greater body length and withers height than calves fed CAN during the preweaning period. Rumen fermentation parameters were also not affected by the treatments, except that calves fed CAN had a higher molar proportion of acetate than calves fed SOY and CASY. Corn grain processing and protein source did not affect glucose, blood urea nitrogen (BUN), or β-hydroxybutyrate (BHB) concentrations, except for the highest blood glucose level observed in the CAN treatment and the highest BUN level observed in the preweaned calves fed SOY. However, a two-way interaction was observed for BHB concentration, suggesting that ground corn grain resulted in higher BHB concentration during the preweaning and postweaning periods than steam-flaked corn. In summary, it is recommended to incorporate canola meal with ground corn or soybean meal with steam-flaked corn in calf starters to enhance calf growth.

## Introduction

Newborn calves lack a functional rumen and need to develop this function, which largely depends on the chemical composition of their diet and its fermentation in the rumen^1^. Discussions are ongoing regarding the use of certain processing methods or forms of calf starter to enhance growth performance in dairy calves^1^. Coarse or fine grinding is a common and cost-effective grain processing method that reduces the particle size of feed ingredients^1^. Many textured calf starters consist of steam-flaked, steam-rolled, coarse-rolled, or whole grains in combination with a pelleted additive. Research shows that textured starters containing steam-flaked corn grain improved calf performance compared to finely ground^2^ or coarsely ground corn grain^3^. Heat treatments like steam flaking can enhance starch digestibility compared to mechanically processed corn, such as milling and dry rolling^4,5^. Steam flaking increases the surface area of the corn and facilitates greater microbial binding and digestion of starch granules, thereby increasing energy and microbial protein availability^6^. Mojahedi et al.^7^ found in a study with dairy calves that feeding steam-flaked corn increased average daily gain (ADG) compared to cracked corn, but only when combined with alfalfa hay.

Canola meal is a high-quality protein source for animals, containing 38–42% of DM crude protein (CP) compared to 49.9% of DM CP in soybean meal^8–10^. Canola meal is not usually recommended for feeding to dairy calves due to inedible components and nutrient-hostile factors that make it less palatable and digestible^11,12^. Some studies have shown that the use of canola meal as a protein source in calf meal may reduce feed intake and nutrient digestibility, especially compared with soybean meal^11,12^, although results are conflicting^13,14^. Canola protein isolate has higher water solubility than soy isolates in all pH ranges, while isolated soy proteins have low solubility in the pH range studied^15–17^. According to a previous study^18^, the partial replacement of canola meal may be an appropriate option, as it was found that replacing 50% of the crude protein (CP) of soybean meal with canola meal had no negative effects on feed intake and growth performance during weaning. However, the complete replacement of soybean meal with canola meal in the starter feed had a negative effect on ADG, feed efficiency (FE), and fecal scores of calves during the preweaning period^18^. A recent study investigated the impact of incorporating canola meal into pelleted starter mixes with similar levels of CP, starch, and neutral detergent fiber (NDF) for Holstein heifers. It was found that up to 60% of the CP of soybean meal can be replaced by canola meal without negative effects on calf body weight, starter intake, ADG, or FE^19^.

Optimizing the balance between dietary protein and energy in the rumen of dairy calves is critical to their growth performance^20,21^. Research has identified an optimal CP-to-energy ratio of 63 g CP/megacalories (Mcal) of metabolizable energy (ME) for weaned calves weighing 60 kg^22^, 52 to 59 g CP/Mcal of ME for calves weighing 95 kg^22^, and 59 g CP/Mcal of ME for dairy heifers weighing 150 kg^23^. Techniques such as grinding, steam flaking, or rolling can affect the surface area and accessibility of starch granules to rumen bacteria, which ultimately affects starch digestion in the rumen of dairy calves^24^. The concentration of RDP in soybean meal and canola meal^25^, as well as the grain processing methods used for calf starters^24^, could greatly affect the nutrient digestibility, feed intake, and growth rate of dairy calves. Therefore, it is critical to understand the intricate interplay between protein sources and grain processing to optimize rumen energy and protein release and utilization in dairy calves. Currently, there is limited information on the protein source in calf starter feeds and the potential effects of grain processing on calf performance and rumen fermentation.

Therefore, the objective of our study was to investigate the interactive effects of 2 different processing methods of corn grain (coarsely ground or steam-flaked) with three protein sources (100% canola meal (CAN), 50% canola meal + 50% soybean meal (CASY), 100% soybean meal (SOY) in the starter feed on growth performance, rumen fermentation, and selected blood metabolites in Holstein dairy calves during pre- and post-weaning period. It was hypothesized that calves fed different rumen starch availability from steam-flaked corn and coarsely ground corn would respond differently depending on nitrogen availability from canola or soybean meal in the starter diet.

## Material and methods

### Animal ethic statement

The experiment was conducted from January and April 2021 in a large commercial dairy farm (Fazil Agri. Animal Production Co., Isfahan, Iran). Ethical approval for all procedures involving animals was obtained from the Animal Care and Use Committee of Isfahan University of Technology (IUT, Iran; IACUC #2020/C05/2) before the start of the study. All methods were performed following the Animal Care and Use Committee of the Iranian Council for Animal Care^26^. The study complies with ARRIVE guidelines for reporting in vivo experiments and all methods were performed following the relevant guidelines and regulations. Experimental research and field studies on plants (either cultivated or wild), including the collection of plant material, must comply with relevant institutional, national, and international guidelines and legislation.

### Animals and management

In this study, seventy-two Holstein calves with an average body weight of 39 ± 3 kg were subjected to six different treatments. Treatments were randomly assigned in a 2 × 3 factorial arrangement, with the physical form of corn grain (coarsely ground vs. steam flaked) and protein sources (canola meal, canola meal + soybean meal, soybean meal) as the two factors. Each treatment included twelve calves (six males and six females). After birth, calves were immediately separated from their dams, weighed, and housed in individual pens (1.2 × 2.5 m) lined with sawdust, which was renewed every morning. Within 1.5 h after birth, calves received 2.5 L of colostrum, while 12 h after the first feeding, 2.5 L were administered. During the first three days of life, the newborn was fed with transition milk. Calves were subjected to a moderate (or restricted) milk feeding method in which they received 5 L/day of whole milk in steel pails twice daily at 0500 and 1400 h from day 3 to 14 and 7 L/day from day 15 to 50 of the study. Following that, 2.5 L of milk was consumed per day until day 56. Milk samples were collected weekly and subsequently analyzed for fat, CP, lactose, and total solids using an infrared spectrophotometer (FOSS milko-scan; FOSS Electric, HillerØd, Denmark). The average composition of the milk supplied was 3.16 ± 0.08% fat, 3.03 ± 0.06% CP, 4.82 ± 0.04% lactose, and 11.9% total solids. Calves were weaned on day 57 and lasted until day 70. During this time, calves had unrestricted access to clean fresh water and starter feed.

### Experimental treatments and chemical analysis

Calves in this study were randomly assigned to one of six feeding treatments, as follows (1) CG-CAN, consisting of coarsely ground corn grain and 100% canola meal; (2) CG-CASY, consisting of coarsely ground corn grain, 50% canola meal, and 50% soybean meal; (3) CG-SOY, consisting of coarsely ground corn grain and 100% soybean meal; (4) SF-CAN, consisting of steam flaked corn grain and 100% canola meal; (5) SF-CASY, consisting of steam flaked corn grain, 50% canola meal and 50% soybean meal; and (6) SF-SOY, consisting of steam-flaked corn grain and 100% soybean meal. All starter diets had the same composition of energy, protein, and other nutrients but differed in the physical form of the corn grain and protein sources. The basal diet in this study contained 5% chopped wheat straw (DM), which is the minimum requirement for a starter diet to prevent a drop in rumen pH^27^. Throughout the experiment, forage samples were collected weekly, stored at − 20 °C, and analyzed for chemical composition. Subsamples of the forage and waste were thoroughly mixed, dried at 55 °C for 48 h, and ground through a 1-mm sieve using a Wiley mill (Ogaw Seiki Co., Ltd., Tokyo, Japan) before chemical analysis for DM^28^, CP^28^, lipids^28^, and NDF content using a heat-stable α-amylase (100 μL/0.5 g of sample) and sodium sulfite^29^. The ingredients and chemical composition of the experimental diets are listed in Table 1. The diets were formulated according to NRC^30^ recommendations.

**Table 1:** Ingredients, chemical composition, and particle size distribution of experimental diets (% of TMR DM).

Item	Coarsely ground			Steam flaked		
	CAN	CASY	SOY	CAN	CASY	SOY
Ingredients						
Wheat straw	5.00	5.00	5.0	5.0	5.0	5.0
Corn grain	44.0	50.5	55.0	44.0	50.5	55.0
Canola meal	43.5	18.5	–	43.5	18.5	–
Soybean meal	–	18.50	32.50	–	18.5	32.5
Calcium carbonate	1.50	1.50	1.50	1.50	1.50	1.50
Dicalcium phosphate	0.50	0.50	0.50	0.50	0.50	0.50
Sodium bicarbonate	1.50	1.50	1.50	1.50	1.50	1.50
Magnesium oxide	0.50	0.50	0.50	0.50	0.50	0.50
Salt	0.50	0.50	0.50	0.50	0.50	0.50
Bentonite	0.50	0.50	0.50	0.50	0.50	0.50
Vitamin premix^a^	1.25	1.25	1.25	1.25	1.25	1.25
Mineral premix^b^	1.25	1.25	1.25	1.25	1.25	1.25
Chemical composition, (g/kg of DM)						
DM	93.50	93.00	93.20	93.45	93.65	93.10
OM	86.51	86.42	86.49	86.55	86.44	86.49
CP	18.51	18.46	18.42	18.54	18.52	18.58
EE	4.30	4.27	3.95	4.28	4.22	3.80
NDF	27.74	20.40	17.71	26.05	24.16	19.42
ADF	11.95	11.55	6.44	12.45	11.36	7.75

*DM* dry matter, *CP* crude protein, *EE* ether extract, *NDF* neutral detergent fiber, *ADF* acid detergent.

Cornprocessing (coarsely ground vs steam flaked) and protein source [100% canola meal (CAN), 50% canola meal + 50% soybean meal (CASY), 100% soybean meal (SOY)].

^a^Vitamin premix: Vit A (IU) = 1,150,000, Vit D3 (IU) = 80,000, Vit E (IU) = 6700, Vit B1(ppm) = 880, Vit B2 (ppm) = 850, Vit B3 (ppm) = 1740, Vit B5 (ppm) = 1346, Vit B6 (ppm) = 873, Vit B9 (ppm) = 77, Vit B12 (ppm) = 9.3, Vit C (ppm) = 16,500, Biotin (ppm) = 13.5, Choline (ppm) = 7500.

^b^Mineral premix: Mg (ppm) = 44,000, Ca (ppm) = 64,000, P (ppm) = 30,000, Na (ppm) = 60,000, Cl (ppm) = 75,000, Fe (ppm) = 10,500, Mn (ppm) = 4000, Zn (ppm) = 4600, Cu (ppm) = 1000, I (ppm) = 25, Co (ppm) = 10, Se (ppm) = 37.

### Grain processing and particle size distribution

Corn grains from the same batch were used for both processing methods. Corn grains were coarsely ground using a hammer mill with a 3-mm sieve (model 5543 GEN, Isfahan Dasht, Isfahan, Iran). Steam-flaked corn (SFC) was prepared using a flaker (Lantus, C22129091, Chavdaneh, Isfahan, Iran) according to the method described by Plascencia and Zinn^31^. The particle size distribution of the experimental feed and its GMPS was measured with dry sieves (ASAE, 1995) using the following sieve sizes: 4.75, 2.36, 1.18, 0.6, 0.3, and 0.15 mm (Table 2).

**Table 2:** Particle size distribution of experimental diets.

g/kg of particles retained on sieve	Steam flaked	Coarsely ground
4.75 mm	19.4 ± 0.80	20.4 ± 0.80
2.36 mm	68.9 ± 0.80	25.0 ± 3.20
1.18 mm	4.0 ± 1.00	11.8 ± 1.00
0.6 mm	3.1 ± 0.60	24.4 ± 1.10
0.3 mm	2.3 ± 0.60	13.6 ± 2.60
0.15 mm	0.9 ± 0.30	3.1 ± 1.00
Pan	0.0 ± 0.0	0.2 ± 0.40
GMPL^a^ mm	2.28 ± 0.05	1.21 ± 0.11

^a^*GMPL* geometric mean particle length; calculated as described by the American Society of Agricultural Engineers (1983).

### Intake and growth performance

To closely monitor calf feed intake, the amount of feed offered was adjusted daily to ensure that 5–10% of the starter feed was not consumed after 24 h. Orts were collected and weighed daily at 0800 h, while feed refusal was recorded at 0730 h. Fresh starter feed was then provided at 0800 h, and water was freely available to calves throughout the experimental period. Calf body weight was measured with an electronic scale at 10-day intervals from day 1 (day 3) to day 70 of the experiment, with weighing occurring before each morning meal to exclude the effects of gastrointestinal status on body weight. Daily body weight gain was calculated by dividing the weight gain achieved over 10 days by 10. To determine the total feed intake DM of each calf during the study, the weights of feed offered and refused were recorded daily. ADG before and after weaning, as well as average total daily gain and FE, was calculated using the following formula: kg BW gain/kg total DM intake (TDMI; starter DM intake + milk DM intake). Finally, body length, heart girth, withers height, hip height, and hip width of all calves were measured on the day of weaning (day 56) and the last day of the study (day 70) according to Lesmeister and Heinrichs^32^.

### Ruminal sampling and chemical analysis

Rumen fluid samples were collected from the animals with a stomach tube connected to a vacuum pump 3 h after the morning feeding between 11:00 and 12:00 on two different days (days 45 and 65). To avoid possible contamination by saliva, the first 50 mL of each sample was discarded. The pH of the first sample was measured with a pH meter (HI 8318; Hanna Instruments, Cluj-Napoca, Romania), which was calibrated before each measurement. Subsequently, the collected rumen fluid samples were filtered through four layers of cheesecloth, and a 10 mL aliquot of each sample was preserved with 2 mL of 25% meta-phosphoric acid and frozen at − 20 °C for analysis of molar volatile fatty acids (VFA). Rumen fluid samples were then thawed and centrifuged at 10,000×*g* and 4 °C for 20 min before VFA analysis. VFA analysis was performed by gas chromatography^33^ using a 0.25 × 0.32 mm, 0.3 µm i.d. fused silica capillary (model no. CP-9002Vulcanusweg 259 a.m., Chrompack, Delft, The Netherlands). The internal standard was crotonic acid, and the carrier gas was nitrogen. The detector and injector temperatures were set at 250 °C, while the initial and final oven temperatures were 55 and 195 °C, respectively.

### Blood sampling and biochemical measurements

Blood samples were collected from dairy calves on days 45 and 65, 3 h after morning feeding. The jugular vein was used for blood collection, and K2 EDTA-containing evacuated tubes were used. Samples were immediately placed on ice and centrifuged at 2850×*g* for 20 min at 4 °C to separate plasma from cells. Then, 1.5 mL of each sample was transferred to 2 mL cryotubes and stored at − 20 °C for subsequent analysis. Plasma concentrations of glucose, urea N, beta-hydroxybutyrate (BHB), and triglycerides were measured using commercial kits (Pars Azmoon Co., Tehran, Iran) and an automated biochemical analyzer (Technicon RA1000; Bayer Corp., Tarrytown, NY, USA) according to the manufacturer's instructions. The results of these measurements were used for further statistical analysis.

### Statistical analysis

Following previously published values,^34,35^ a daily standard deviation of 100 g ADG was assumed, and a difference of 65–75 g per day was considered meaningful. A power test analysis was performed with α = 0.05 and power (1 − β) = 0.80, resulting in an expected sample size of 12 calves per treatment for growth performance. This parameter can be used to most accurately determine power. Data were analyzed using a completely randomized experimental design with a 2 × 3 factorial arrangement of processing of corn grain (coarsely ground and steam-flaked) and protein source (CAN, CASY, and SOY) treatments and analyzed using the MIXED procedure of SAS with analysis of variance (ANOVA). Time served as a repeated measure of starter feed intake, total DMI, ADG, FE, skeletal growth, rumen fermentation traits, and blood metabolites, with the individual calf as the experimental unit. The model included fixed effects of corn grain processing, protein source, time, and their interactions, with the calf included as a random effect. The main effects of corn grain processing, protein source, and interactions were tested using ANOVA. Calf sex was assessed as a fixed factor but excluded from the final model due to its lack of significance. A type 1 autoregressive covariance structure was selected as the best fit based on the Bayesian information criterion after testing three variance–covariance structures (type 1 autoregressive, compound symmetry, and Toeplitz). Residuals were tested for normality using the Shapiro–Wilk statistic and the UNIVARIATE procedure in SAS, as well as the Levenes test for homogeneity of variance and quantile–quantile plots for visual assessment. Data that did not meet the assumptions for the normality of the residuals were log-transformed (base 10). As a result of the log transformation, the distribution of the data was retested and confirmed to be normally distributed. In the analysis of weaning and final BW (d 56 and 70), the initial BW was used as a covariate. A Turkey–Kramer adjustment was applied to account for multiple comparisons. The significance threshold was set at *P* ≤ 0.05, and trends were explained at 0.05 < *P* ≤ 0.10.

## Results

### Intake and growth performance

**Figure 1:**
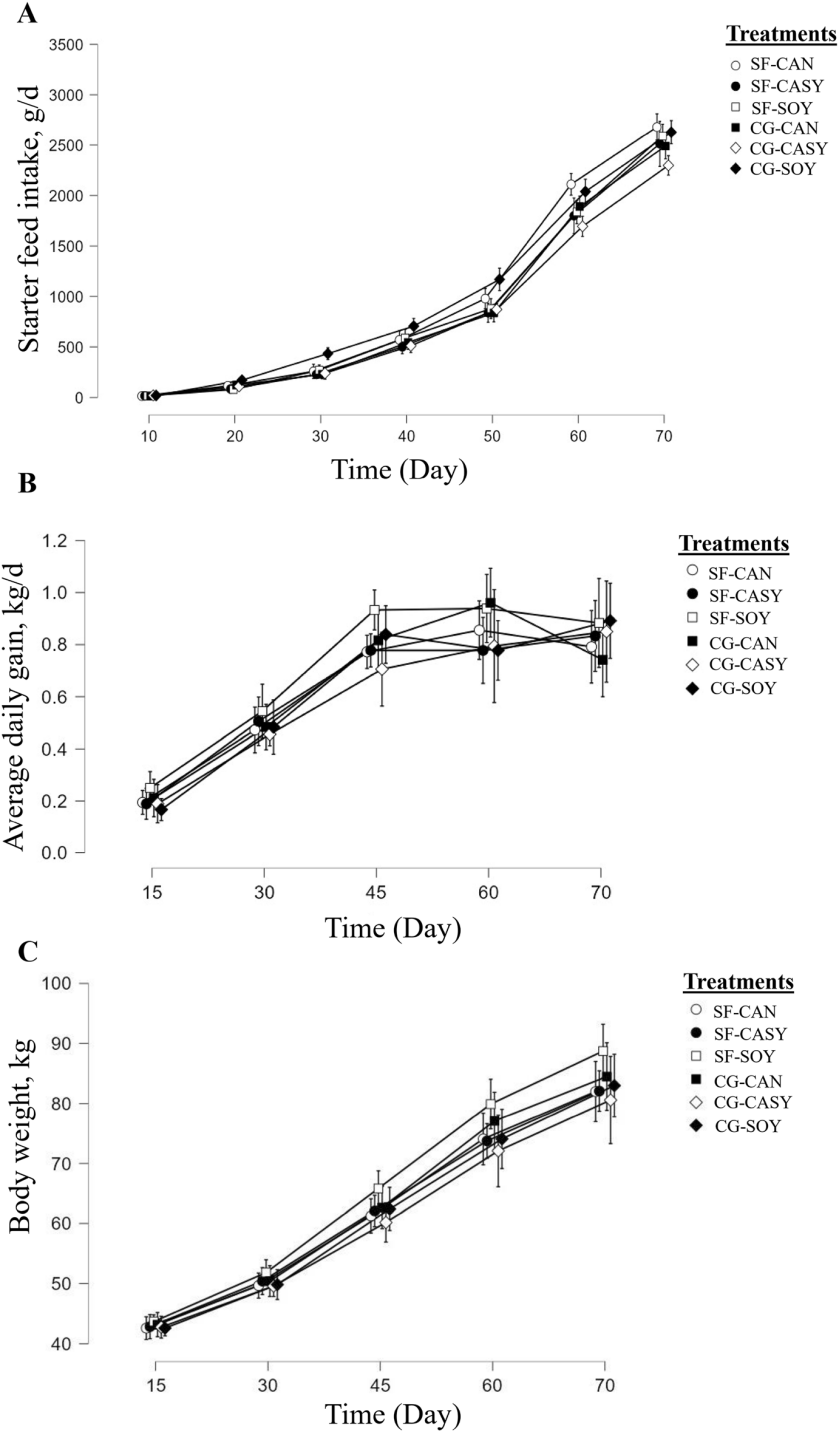
Mean (**A**) starter feed intake, (**B**) average daily gain, and (**C**) body weight of calves with different birth body weights (n = 13 per treatment) fed different treatments. The treatments included: (1) coarsely ground corn grain + 100% canola meal (CG-CAN), (2) coarsely ground corn grain + 50% canola meal + 50% soybean meal (CG-CASY), (3) coarsely ground corn grain + 100% soybean meal (CG-SOY), (4) steam-flaked corn grain + 100% canola meal (SF-CAN), (5) steam-flaked corn grain + 50% canola meal + 50% soybean meal (SF-CASY), and (6) steam-flaked corn grain + 100% soybean meal (SF-SOY). Data are presented as mean ± SEM.

Data for dry matter intake (DMI), starter feed intake (Fig. 1A), ADG (Fig. 1B), BW (Fig. 1C), and FE are shown in Table 3. The greatest feed intake and DMI were observed in the post-weaning period and throughout the period for calves fed CG-CAN and SF-SOY diets, demonstrating the interaction between the corn grain processing method and protein source in the current study (*P* ≤ 0.05). In the preweaning period, feed intake was greater when calves were fed CG-CAN, and the interaction between the corn grain processing method and protein source tended to be significant (*P* = 0.08). The lowest (*P* ≤ 0.05) feed intake was observed at SF-CASY during the post-weaning period and overall. The different processing methods for corn grain had no effect on feed intake, ADG, and FE in the current study. The ADG in the preweaning period (*P* = 0.02) and the total experimental period (*P* = 0.01) was greater in calves fed SF-SOY, followed by those on CG-CAN, showing the interaction between the corn grain processing method and protein source. The highest ADG was obtained at SOY and then at CAN, and the lowest was obtained at CASY in the pre-weaning period (*P* = 0.02) and the total period (*P* = 0.05). The body weight in pre-weaning (*P* = 0.05), post-weaning (*P* = 0.02), and total period (*P* = 0.03) were higher in SF-SOY and then CG-CAN treatment, so the interaction between the corn grain processing method and protein source was meaningful. The greatest BW was in SOY and CAN (*P* = 0.04). A 2-way interaction was observed between protein source and time to BW (*P* < 0.01), suggesting that soybean meal had a greater effect on BW during the preweaning and total periods. A 3-way interaction was found between corn grain processing method, protein source, and time with respect to BW, indicating, that SF-SOY and then CG-CAN had a greater effect on BW during the preweaning (*P* = 0.04), postweaning (trend, *P* = 0.08), and total periods (*P* < 0.01). An interaction was observed between the corn grain processing method and protein source for FE to be improved during the preweaning period (*P* = 0.03) and the entire period (*P* = 0.01) in calves fed CG-CAN and SF-SOY. No 3-way interaction was found between corn grain processing method, protein source, and time with respect to starter intake, DMI, FE, and ADG.

**Table 3:** Interaction between corn grain processing (coarsely ground and steam flaked) and protein source [100% canola meal (CAN), 50% canola meal + 50% soybean meal (CASY), 100% soybean meal (SOY)] on starter intake, DMI, ADG, BW and FE of dairy calves (n = 12 calves per treatment).

Item	Coarsely ground			Steam flaked			SEM	P-value^1^			T	PR × T	P × T	PR × P × T
	CAN	CASY	SOY	CAN	CASY	SOY		PR	P	PR × P				
Total DMI, kg/day														
Pre-weaning (day 3 to 56)	1.38	1.25	1.29	1.28	1.26	1.47	0.07	0.58	0.14	0.10	< 0.01	0.88	0.25	0.67
Post-weaning (day 57 to 70)	2.29^a^	2.07^b^	2.01^b^	2.05^b^	1.82^c^	2.17^a,b^	0.10	0.16	0.06	0.05	< 0.01	0.79	0.44	0.72
Overall (day 3 to 70)	1.68^a^	1.52^b^	1.53^b^	1.53^b^	1.45^b,c^	1.71^a^	0.07	0.77	0.08	0.04	< 0.01	0.19	0.16	0.75
Starter intake, kg/day														
Pre-weaning (day 3 to 56)	0.74	0.59	0.64	0.62	0.59	0.81	0.06	0.70	0.10	0.08	< 0.01	0.93	0.14	0.67
Post-weaning (day 57 to 70)	2.29^a^	2.07^b^	2.01^b^	2.05^b^	1.82^c^	2.17^a,b^	0.10	0.16	0.06	0.05	< 0.01	0.79	0.44	0.72
Overall (day 3 to 70)	1.26^a^	1.08^b^	1.10^b^	1.09^b^	1.01^b,c^	1.27^a^	0.06	0.68	0.07	0.03	< 0.01	0.29	0.17	0.74
BW, kg														
Pre-weaning (day 3 to 56)	57.55	55.10	56.31	55.58	56.22	59.86^a^	1.13	0.33	0.10	0.05	< 0.01	0.57	< 0.01	0.04
Post-weaning (day 57 to 70)	82.61^b^	76.41^c^	78.83^c^	78.12^c^	78.00^c^	86.45^a^	2.13	0.36	0.04	0.02	< 0.01	0.88	0.43	0.08
Overall (day 3 to 70)	65.77^a,b^	62.20^c^	63.78^c^	63.09^c^	63.48^c^	68.63^a^	1.40	0.32	0.06	0.03	< 0.01	0.83	< 0.01	< 0.01
ADG, kg/day														
Pre-weaning (day 3 to 56)	0.63^b^	0.53^d^	0.58^c^	0.54^d^	0.56^c,d^	0.67^a^	0.03	0.67	0.02	0.02	< 0.01	0.40	0.28	0.84
Post-weaning (day 57 to 70)	0.96	0.84	0.82	0.87	0.82	0.99	0.05	0.65	0.27	0.07	0.86	0.80	0.43	0.43
Overall (day 3 to 70)	0.73^b^	0.63^d^	0.66^c^	0.65^c^	0.65^c^	0.78^a^	0.03	0.59	0.05	0.01	< 0.01	0.76	0.51	0.80
FE														
Pre-weaning (day 3 to 56)	0.44^a^	0.40^b^	0.42^a,b^	0.41^b^	0.42^a,b^	0.44^a^	0.01	0.63	0.22	0.03	< 0.01	0.69	0.89	0.32
Post-weaning (day 57 to 70)	0.45	0.41	0.44	0.44	0.47	0.45	0.02	0.25	0.96	0.17	< 0.01	0.63	0.91	0.19
Overall (day 3 to 70)	0.44^a^	0.40^b^	0.42^a,b^	0.42^a,b^	0.44^a^	0.44^a^	0.01	0.23	0.29	< 0.01	< 0.01	0.81	0.98	0.30

*DMI* dry matter intake, *BW* body weight, *ADG* average daily gain, *FE* feed efficiency (kg of BW gain/kg of total DMI).

^1^Statistical comparisons: *PR* processing corn grain, *P* Protein source, *T* Time, *PR* × *P* interaction between processing corn grain and protein source.

^a–^^d^Means within a row with different superscripts letters are significantly different (*P* < 0.05).

The term "Overall" refers to the mean values derived from analyzing data using a repeated measures approach across all measured time points.

### Skeletal growth parameters

Body length, withers height, heart girth, hip width, and hip height were not affected by the corn grain processing method and interaction (Table 4). Calf body length and wither height were greater in the preweaning period in the SOY and CASY treatments compared with the CAN treatment (*P* < 0.05). The greatest hip height was observed in the SOY and CASY treatment in the preweaning period, which tended to be significant (*P* = 0.08). No 3-way interaction was observed between the corn grain processing method, protein source, and time concerning skeletal growth parameters.

**Table 4:** Interaction between corn grain processing (coarsely ground and steam flaked) and protein source (100% canola meal (CAN), 50% canola meal + 50% soybean meal (CASY), 100% soybean meal (SOY)) on skeletal growth of dairy calves (n = 12 calves per treatment).

Item	Coarsely ground			Steam flaked			SEM	*P*-value^1^			T	PR × T	P × T	PR × P × T
	CAN	CASY	SOY	CAN	CASY	SOY		PR	P	PR × P				
Skeletal growth, cm														
Body length														
Day 56	45.63	45.97	47.43	44.62	47.31	47.35	0.85	0.90	0.03	0.38				
Day 70	52.45	52.60	52.82	50.73	54.13	53.82	1.04	0.75	0.15	0.25				
Overall	49.29	49.45	50.33	47.50	50.37	50.50	0.91	0.75	0.07	0.31	< 0.01	0.66	0.61	0.59
Wither height														
Day 56	88.98	89.44	90.30	88.97	90.73	90.88	0.67	0.26	0.05	0.62				
Day 70	93.22	93.32	94.09	93.39	94.31	94.56	0.66	0.31	0.32	0.82				
Overall	91.20	92.04	91.62	91.04	92.62	92.58	0.69	0.42	0.18	0.71	< 0.01	0.86	0.51	0.91
Heart girth														
Day 56	96.22	95.55	96.86	95.94	96.55	98.27	0.83	0.29	0.12	0.57				
Day 70	102.12	100.96	101.92	101.75	101.62	103.79	0.71	0.22	0.09	0.30				
Overall	99.20	98.29	99.25	98.70	99.12	101.21	0.76	0.22	0.11	0.28	< 0.01	0.94	0.44	0.63
Hip width														
Day 56	26.37	26.25	26.15	26.30	26.75	26.82	0.37	0.24	0.88	0.59				
Day 70	28.43	28.05	28.15	27.99	28.53	28.98	0.36	0.34	0.60	0.20				
Overall	27.45	27.25	27.16	27.08	27.54	27.91	0.35	0.44	0.74	0.28	< 0.01	0.67	0.65	0.66
Hip height														
Day 56	91.09	91.38	92.16	91.05	92.90	93.05	0.70	0.17	0.08	0.54				
Day 70	95.34	95.17	95.98	95.51	96.29	96.68	0.67	0.23	0.41	0.78				
Overall	93.20	94.04	93.70	93.04	94.70	94.62	0.70	0.41	0.16	0.72	< 0.01	0.74	0.45	0.86

^1^Statistical comparisons: *PR* processing corn grain, *P* Protein source, *T* Time, *PR* × *P* interaction between processing corn grain and protein.

The term "Overall" refers to the mean values derived from analyzing data using a repeated measures approach across all measured time points, namely day 56 and day 70.

#### Rumen fermentation

The results for the rumen fermentation profile are shown in Table 5. For pH and rumen fermentation profile, no interaction was observed between the corn grain processing method and protein sources. The treatments did not significantly affect rumen pH, total VFA, propionate, butyrate, iso-butyrate, valerate, and iso-valerate molar proportions. The molar proportion of acetate in the rumen of calves fed CAN was higher on days 45 and 65 (trend, *P* = 0.08) than those fed SOY and CASY. Rumen molar proportions of acetate (*P* < 0.05), propionate (*P* = 0.01), and butyrate (*P* = 0.01) were significantly affected by time of sampling. The molar proportions of acetate and butyrate were greater on day 65 compared to day 45, while the molar proportion of propionate in the rumen was lower on day 65 compared to day 45. No 3-way interactions were detected between the corn grain processing method, protein source, and time concerning rumen fermentation parameters.

**Table 5:** Interaction between corn grain processing (coarsely ground and steam flaked) and protein source (100% canola meal (CAN), 50% canola meal + 50% soybean meal (CASY), 100% soybean meal (SOY)) on ruminal pH and VFA of dairy calves (n = 12 calves per treatment).

Item	Coarsely ground			Steam flaked			SEM	*P*-value^1^			T	PR × T	P × T	PR × P × T
	CAN	CASY	SOY	CAN	CASY	SOY		PR	P	PR × P				
Ruminal pH														
Day 45	6.75	6.5	6.36	6.89	6.57	6.78	0.16	0.14	0.19	0.56				
Day 65	6.7	6.63	6.26	6.34	6.68	6.61	0.17	0.92	0.44	0.13				
Overall	6.73	6.57	6.31	6.61	6.63	6.7	0.12	0.27	0.40	0.13	0.29	0.33	0.22	0.54
Individual VFA (mol/100 mol)														
Acetate														
Day 45	51.9	46.78	48.1	49.9	46.95	47.94	1.58	0.62	0.05	0.76				
Day 65	55.77	51.41	49.76	50.56	51.46	47.15	1.20	0.12	0.08	0.43				
Overall	53.83	49.09	48.9	50.23	49.2	47.54	1.02	0.16	0.05	0.20	0.05	0.43	0.4	0.86
Propionate														
Day 45	37.33	42.11	38.36	39.67	41.15	39.67	1.88	0.56	0.22	0.67				
Day 65	31.12	36.26	34.7	34.19	36.2	40.14	2.54	0.18	0.17	0.56				
Overall	34.22	39.18	36.53	36.92	38.68	39.91	1.35	0.12	0.15	0.32	0.01	0.51	0.43	0.86
*Iso-*butyrate														
Day 45	0.34	0.34	0.45	0.31	0.26	0.43	0.07	0.42	0.22	0.95				
Day 65	0.44	0.46	0.47	0.43	0.29	0.41	0.16	0.52	0.92	0.90				
Overall	0.39	0.4	0.47	0.36	0.27	0.43	0.08	0.34	0.57	0.86	0.44	0.8	0.86	0.95
Butyrate														
Day 45	7.16	6.94	8.68	7.36	7.34	8.95	0.90	0.70	0.14	0.99				
Day 65	9.75	7.62	10.61	11.5	9.22	8.83	1.21	0.60	0.21	0.27				
Overall	8.45	7.28	9.64	9.42	8.27	8.88	0.65	0.45	0.13	0.32	0.01	0.86	0.31	0.51
*Iso*-valeric														
Day 45	0.49	0.53	0.65	0.40	0.44	0.62	0.11	0.32	0.10	0.90				
Day 65	0.56	0.69	0.84	0.65	0.48	0.72	0.22	0.69	0.63	0.77				
Overall	0.52	0.61	0.75	0.53	0.45	0.67	0.11	0.42	0.18	0.76	0.19	0.98	0.99	0.81
Valeric														
Day 45	2.78	3.3	3.76	2.36	3.86	2.39	0.60	0.41	0.27	0.29				
Day 65	2.36	3.56	3.62	2.67	2.35	2.75	0.68	0.29	0.61	0.51				
Overall	2.59	3.44	3.71	2.54	3.13	2.57	0.43	0.16	0.22	0.44	0.63	0.83	0.72	0.36
Total VFA, mmol/L														
Day 45	72.21	81.68	91.54	78.44	78.89	80.01	7.99	0.68	0.43	0.54				
Day 65	78.86	83.18	72.71	58.8	70.14	70.75	8.37	0.25	0.81	0.76				
Overall	75.48	82.43	82.13	68.62	74.51	75.38	6.90	0.21	0.55	0.99	0.21	0.48	0.78	0.51

^1^Statistical comparisons: *PR* processing corn grain, *T* time, *P* Protein source, *PR* × *P* interaction between processing corn grain and protein source.

The term "Overall" refers to the mean values derived from analyzing data using a repeated measures approach across all measured time points, namely day 45 and day 65.

#### Blood parameters

Data for glucose, triglycerides (TG), blood urea nitrogen (BUN), and BHB concentration are shown in Table 6. For glucose, TG, and BUN, no interaction was observed between the corn grain processing method and protein source. The greatest blood glucose concentration was observed in the CAN treatment (*P* = 0.01). In the pre-weaning, the BUN concentration was greater when calves were fed SOY than the other treatments (*P* < 001). The greatest BHB concentration in the preweaning period was observed in calves fed SF-SOY (*P* = 0.01) and their interaction was observed in the current study (*P* = 0.01), but in the post-weaning period was found for CG-CAN (*P* = 0.03). We did not detect a 3-way interaction between the corn grain processing method, protein source, and time when glucose, BUN, and BHB were considered. A 3-way interaction between corn grain processing method, protein source, and time was observed for TG concentration (Table 6; *P* = 0.03), suggesting that corn processing and protein source had a positive effect on TG concentration during the postweaning period. In addition, a 2-way interaction between corn grain processing method and time was observed for BHB concentration (*P* < 0.01), indicating that BHB concentration was positively influenced in the coarsely ground compared with steam flaked during the preweaning and postweaning periods.

**Table 6:** Interaction between corn grain processing (coarsely ground and steam flaked) and protein source [100% canola meal (CAN), 50% canola meal + 50% soybean meal (CASY), 100% soybean meal (SOY)]on blood metabolites of dairy calves (n = 12 calves per treatment).

Item	Coarsely ground			Steam flaked			SEM	*P*-value^1^			T	PR × T	P × T	PR × P × T
	CAN	CASY	SOY	CAN	CASY	SOY		PR	P	PR × P				
Glucose, mg/dL														
Day 45	88.33	86.83	94.66	80.83	88.66	94.00	3.27	0.43	0.01	0.35				
Day 65	71.00	72.83	65.16	76.50	78.83	69.33	7.15	0.37	0.46	0.99				
Overall	79.66	79.83	79.91	78.66	83.75	81.66	4.32	0.66	0.82	0.85	< 0.01	0.20	0.04	0.78
Triglycerides, mg/dL														
Day 45	52.00	60.33	46.00	75.33	50.50	60.83	9.17	0.21	0.50	0.18				
Day 65	41.16	26.83	27.33	28.16	29.66	25.66	4.65	0.30	0.19	0.23				
Overall	46.58	43.58	36.66	51.75	40.08	43.25	5.70	0.55	0.24	0.63	< 0.01	0.08	0.96	0.03
BUN, mg/dL														
Day 45	15.83	12.83	19.33	16.00	12.83	21.50	1.68	0.57	0.01	0.77				
Day 65	26.83	24.83	27.83	24.83	24.00	29.00	2.55	0.79	0.29	0.82				
Overall	21.33	18.83	23.58	20.41	18.41	25.25	1.48	0.92	< 0.01	0.65	< 0.01	0.60	0.53	0.97
BHB, mmol/L														
Day 45	0.25^b^	0.16^d^	0.23^b^	0.22^b,c^	0.20^b,c^	0.37^a^	0.02	0.01	0.01	0.01				
Day 65	0.51	0.39	0.43	0.37	0.27	0.44	0.04	0.03	0.03	0.22				
Overall	0.38^a^	0.27^c,d^	0.33^b^	0.29^b,c^	0.24^d^	0.40^a^	0.02	0.41	< 0.01	< 0.01	< 0.01	< 0.01	0.36	0.87

*BUN* blood urea nitrogen, *BHB* beta-hydroxybutyrate.

^1^Statistical comparisons: *PR* processing corn grain, *P* Protein source, *T* time, *PR* × *P* interaction between processing corn grain and protein source.

^a–d^Means within a row with different superscripts letters are significantly different (*P* < 0.05).

The term "Overall" refers to the mean values derived from analyzing data using a repeated measures approach across all measured time points, namely day 45 and day 65.

## Discussion

### Growth performance and rumen fermentation

In the present experiment, the greatest feed intake and DMI were observed in the post-weaning period for CG-CAN and in the total period for SF-SOY treatment showing the interaction between the corn grain processing method and protein source in the current study. Makizadeh et al.^36^ reported no difference between ground corn grain and steam-flaked corn grain for feed intake. Grinding grains reduces ruminal pH and reduces feed intake which is partly related to small particle size and increased fermentation rate^37,38^. Zhang et al.^39^ suggested that there was no difference between processing methods (coarsely ground, steam flaked, and extrusion) for feed intake and DMI. According to the results of this study, the effect of protein sources in the post-weaning period and the whole period was meaningful. In a study by Hadam et al.^18^, it was shown that the replacement of soybean meal with canola meal (50% replacement) did not differ among treatments for DMI and feed intake. In other studies, canola meal can replace up to 60% of the CP provided by soybean meal without affecting the starter intake and growth of calves^19^. Although previous studies, when fully replacing soybean meal with canola meal include decreased starter intake^40^, no studies have reported how canola meal affects calf response. Hadam et al.^18^ reported replacement of soybean meal with canola meal reduced only pre-weaning feed intake (no reduction in post-weaning feed intake was observed) but in complete replacement, feed intake decreased in the total period, which has probably been associated with decreased starch concentrations and increased NDF and ADF concentrations.

The interaction between corn grain processing and protein source in the post-weaning period and the total period was found, which probably indicates the simultaneous availability of starch and protein in calf performance. Chishti et al.^41^ reported weaned dairy calves fed high starch (HS) diets improved DM digestibility and FE by increasing starch digestibility. However, calves fed high protein (HP) diets improved DM digestibility and FE by increasing NDF and ADF digestibility. Increasing starch in the calf diet can decrease rumen pH and negatively affect fiber digestibility. Feeding an HP diet with an HS diet increases rumen pH, and total-tract digestion of DM, NDF, and ADF, so an interaction seems to exist between dietary protein and starch in the rumen of weaned dairy calves. In the present experiment, ADG, BW, FE, and skeletal growth of calves were not affected by corn grain processing. Some previous studies reported no effect of corn grain processing on ADG, BW, and FE of calves^39^, while the study by Makizadeh et al.^36^ reported that ADG, BW, and FE were higher when calves fed steam flaked corn grain were compared with ground corn grain, but no differences were found between treatments for skeletal growth. It is important to note that in our study, we implemented a moderate (or restricted) feeding method that provided a low amount of milk to the calves. This approach may have contributed to the lower ADG observed during the preweaning period in our study.

The present study investigated the effect of protein sources on various parameters such as ADG, BW, body length, waist height, and hip height and results showed that protein sources had a significant effect on these parameters. Makizadeh et al.^36^ reported the effects of protein content on calf performance but found no significant effects on ADG or FE. Haddam et al.^18^ indicated that replacing soybean meal with canola meal could reduce ADG and FE, but replacing soybean meal with canola meal up to 50% had no negative effect on calf performance. This is consistent with other studies that found no significant differences in ADG, BW, and FE between treatments when soybean meal was replaced with canola meal^19^. In the present study, an interaction between corn grain processing and protein source was also observed in ADG, BW, and FE, suggesting that simultaneous access to energy and protein affects calf performance. In the study, no significant increase in ADG in response to energy intake (corn grain) was observed when dietary CP content was between 5 and 10%, suggesting that when protein limits growth, calves do not respond to higher energy intakes. However, a significant increase occurred when calves received protein supplementation (N 15% of total CP in the diet). These results seem to support the existence of protein- and energy-dependent growth phases. The relationship between energy intake and amino acid (AA) requirements is critical for calf growth. The higher the energy intake, the greater the growth potential, resulting in higher amino acid requirements. In addition, amino acid requirements can be expressed concerning energy intake because the relationship between energy intake and the rate of protein deposition is linear^42^. Grain processing provides starch availability and ultimately energy for calves. The results of this study provide important insight into the interaction between protein source and corn processing method (starch availability) and suggest that a balanced diet with both starch and protein is essential for optimal calf growth and performance.

The results of the study show that most rumen fermentation parameters such as rumen pH, total VFA, molar proportions of propionate, butyrate, iso-butyrate, valerate and iso-valerate were not significantly affected by the administered treatments. In the current study, no interaction was found between corn grain processing method and protein source for pH and rumen fermentation profile. However, the molar proportion of acetate in the rumen of calves fed CAN was significantly higher than that of calves fed SOY and CASY, which can be attributed to the higher NDF content in the diet of CAN. This result is consistent with a previous report by Makizadeh et al.^36^ that reported no interaction between corn grain processing and starter protein content in rumen fermentation variables. Recent studies have also shown that replacement of soybean meal with canola meal in the diets of dairy cows did not show significant differences between treatments in digestibility of NDF, ADF, and organic matter, as well as molar content of acetate, propionate, butyrate, isobutyrate, valerate, isovalerate, and total VFA^43,44^. Burakowska et al.^40^, the use of canola meal instead of soybean meal in calf starters may have positive effects on calf performance by balancing starch and NDF but there was no difference in the concentration of VFA between treatments. In this study, the molar proportions of acetate and butyrate in the rumen of calves increased and the molar proportions of propionate decreased in postweaning than in preweaning period, which may be related to changes in starter feed intake and rumen microbial population^45^. As a result of weaning, a decrease in *Succiniclasticum* in the rumen of calves was reported by Dias et al.^46^. *Succiniclasticum* is a genus capable of producing propionate from lactic acid^47,48^, and this changes in rumen microbial population may be related to the lower rumen molar proportion of propionate after weaning. However, rumen microbiota was not analysed in our study, and further studies are needed to investigate the microbial changes during the pre- and post-weaning period.

### Plasma metabolites

Blood urea nitrogen is an important indicator of rumen nitrogen uptake because it correlates positively with rumen NH_3_–N concentration^49^. BUN is also an important indicator of nitrogen efficiency because it has a strong linear relationship with urinary nitrogen excretion^20^. In the current study, corn processing did not affect concentrations of BUN. However, calves fed starter diets with different protein sources showed significant differences in BUN concentrations. This suggests that the higher BUN concentrations in calves fed soybean meal compared to canola meal may be due to increased rumen NH_3_–N concentrations. This result is inconsistent with a previous report by Makizadeh et al.^36^ who reported that the concentration of BUN before weaning was significantly lower in calves fed steam-flaked corn than in calves fed ground corn.

Considering that canola meal may have higher levels of rumen undegraded protein (RUP) than soybean meal^50^, shifting the site of digestion to the post-ruminal regions of the gastrointestinal tract may limit the digestibility of the entire tract CP in calves. In line with our results, Burakowska et al.^19^ reported that increasing the proportion of canola meal up to 60% of the CP of soybean meal decreased plasma urea concentration. It was reported that use of canola meal as a substitute for soybean meal did not affect the activity of intestinal proteolytic brush border enzymes in calves^40^, and did not affect pancreatic secretion^12^. Thus, replacing soybean meal with canola meal due to its high RUP content reduces the availability of amino acids to rumen bacteria, resulting in a decrease in plasma urea concentration^40^. The use of canola meal instead of soybean meal in the diet of lactating cows did not affect glucose and BHB concentrations^51^ and improved nitrogen use efficiency by decreasing BUN concentrations, increasing branched amino acid concentrations, and increasing metabolizable protein supply to the gut^43^. Overall, the results of this study indicate that corn grain processing methods and protein sources play a critical role in calf performance. These factors play a critical role in calf growth and development and should therefore be considered in dairy calf nutrition practices.

## Conclusions

In summary, this study highlights the importance of corn grain processing methods and protein sources on calf performance. Calves fed coarsely ground corn grain and canola meal and calves fed steam-flaked corn and soybean meal performed better. However, the interaction between corn processing method and protein source did not significantly affect skeletal growth or rumen fermentation parameters. According to the results, it is recommended to use canola meal as the preferred protein source when ground corn grain is the primary energy source in the starter feed. Conversely, it is recommended to include soybean meal in the starter feed when steam-flaked corn is the primary energy source to promote calf growth.

## Data Availability

The datasets used and/or analyzed during the current study are available from the corresponding author on reasonable request.
